# Plant “intelligence” changes nothing

**DOI:** 10.15252/embr.202050395

**Published:** 2020-04-16

**Authors:** David G Robinson, Andreas Draguhn, Lincoln Taiz

**Affiliations:** ^1^ Centre for Organismal Studies University of Heidelberg Heidelberg Germany; ^2^ Institut für Physiologie und Pathophysiologie, Medizinischen Fakultät University of Heidelberg Heidelberg Germany; ^3^ Department of Molecular, Cellular and Developmental Biology University of California Santa Cruz Santa Cruz CA USA

**Keywords:** Ecology, Plant Biology

## Abstract

A comment on “Plants, climate and humans”.

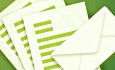

We view the publication of the article by Baluška & Mancuso in *EMBO reports* 1 with considerable scepticism. The authors, principal advocates of the plant neurobiology concept, have tried in numerous articles to disseminate the notion that plants are intelligent organisms that make conscious decisions, based on hypothesized cognitive acts. In several papers, we have taken great pains to separate fact from fiction in regard to “plant intelligence” 2, 3, 4. We conclude that there is no solid scientific evidence to support the claims that plants possess neurons or have the equivalent of a brain, feel pain or contain a memory. Words like “smart” and “intelligent” are now being used rather loosely as in “smart phones” and “intelligent machines,” and it is only in this very broad sense that plants can be considered “intelligent”.

Part of the confusion stems from the use of the misleading term “ecological strategy”. Plants in an ecosystem do not stand around thinking about what their “ecological strategy” will be, and then act on their decisions, as in game theory. Most ecologists understand that “ecological strategy” is a misleading teleological shorthand for evolved adaptive behaviour determined by natural selection. Baluška & Mancuso seem to have taken the term “ecological strategy” literally in their ideas about plants. In short, plants are not “conscious organisms” that make conscious strategic decisions. If current climate models are incomplete (as they most assuredly are), it is not because they are overlooking plant intelligence or consciousness. It is because we still have much to learn about the adaptive responses and interactions of plants in the biosphere.

Attempts to humanize plants may be in line with current trends towards rampant anthropomorphism in biology, but paint a highly distorted picture of plant life. The present article in *EMBO Reports* adds an extra dimension to the apparent cognitive and social abilities of plants: sentences like “A new view of higher plants as cognitive and intelligent organisms that actively manipulate their environment to serve their needs” and “Humans are not excluded from plants’ manipulative behaviour…” appeal to psychological and neurobiological concepts of social cognition without providing empirical basis for such a far‐reaching proposal. We agree that plants make an indispensable contribution to homeostasis in the biosphere and that they are highly complex organisms featuring multiple interactions with their environment. We maintain, however, that the plant science community is not benefited by the approach taken by plant neurobiologists and that it is highly misleading to the general public.
